# Male predominance in reported Visceral Leishmaniasis cases: Nature or nurture? A comparison of population-based with health facility-reported data

**DOI:** 10.1371/journal.pntd.0007995

**Published:** 2020-01-29

**Authors:** Kristien Cloots, Sakib Burza, Paritosh Malaviya, Epco Hasker, Sangeeta Kansal, Guy Mollett, Jaya Chakravarty, Nurpur Roy, Bibek Kumar Lal, Suman Rijal, Shyam Sundar, Marleen Boelaert

**Affiliations:** 1 Department of Public Health, Institute of Tropical Medicine, Antwerp, Belgium; 2 Médecins Sans Frontières, New Delhi, India; 3 Department of Medicine, Banaras Hindu University, Varanasi, India; 4 Faculty of Infectious and Tropical Diseases, London School of Hygiene and Tropical Medicine, London, United Kingdom; 5 National Vector Borne Disease Control Program, Ministry of Health & Family Welfare, New Delhi, India; 6 Epidemiology and Disease Control Division, Ministry of Health, Kathmandu, Nepal; 7 Drugs for Neglected Diseases initiative, New Delhi, India; Universidade do Estado do Rio de Janeiro, BRAZIL

## Abstract

**Background:**

Bangladesh, India, and Nepal aim for the elimination of Visceral Leishmaniasis (VL), a systemic parasitic infectious disease, as a public health problem by 2020. For decades, male patients have comprised the majority of reported VL cases in this region. By comparing this reported VL sex ratio to the one observed in population-based studies conducted in the Indian subcontinent, we tested the working hypothesis that mainly socio-cultural gender differences in healthcare-seeking behavior explain this gender imbalance.

**Methodology/Principal findings:**

We compared the observed sex ratio of male versus female among all VL cases reported by the health system in Nepal and in the two most endemic states in India with that observed in population-based cohort studies in India and Nepal. Also, we assessed male sex as a potential risk factor for seroprevalence at baseline, seroconversion, and VL incidence in the same population-based data. The male/female ratio among VL cases reported by the health systems was 1.40 (95% CI 1.37–1.43). In the population cohort data, the age- and study site-adjusted male to female risk ratio was 1.27 (95% CI 1.08–1.51). Also, males had a 19% higher chance of being seropositive at baseline in the population surveys (RR 1.19; 95% CI 1.11–1.27), while we observed no significant difference in seroconversion rate between both sexes at the DAT cut-off titer defined as the primary endpoint.

**Conclusions/Significance:**

Our population-based data show that male sex is a risk factor for VL, and not only as a socio-cultural determinant. Biological sex-related differences likely play an important role in the pathogenesis of this disease.

## Author summary

Visceral Leishmaniasis (VL) is a parasitic disease that is lethal if not treated timely and mainly affects impoverished populations. Bangladesh, India, and Nepal have targeted the elimination of this disease as a public health problem by 2020. The majority of VL patients attending the health services are male, and this is usually attributed to unequal access to health care for men and women in this sociocultural context. We analyzed two large datasets obtained in population surveillance projects, including regular door-to-door screening for VL, in India and Nepal. Thereby we minimized any potential differences in access to health care between both sexes, as every suspect VL case occurring in the community received a full diagnostic work-up, and the research project facilitated transport to treatment centers if needed. By comparing the observed sex ratio in the health services records with those of the population surveillance records, we aimed to reach meaningful conclusions about the pathway through which male gender exerts its leverage for increasing VL risk: socio-cultural determinants blocking women from accessing care (nurture) or biological factors (nature) making men more vulnerable to VL. Because in the population-based age adjusted-data, male VL cases were significantly more frequent than female, our findings strongly suggest that in the Indian subcontinent, biological differences between men and women play a more critical role in the pathogenesis of VL than previously assumed, and the observed male predominance in VL cases in health services cannot be explained by socio-cultural factors only. Moreover, data show that above the age of 14 years, males are seropositive more often than females and are at higher risk to develop VL disease.

## Introduction

Visceral leishmaniasis (VL) is on the verge of being eliminated as a public health problem in Bangladesh, India, and Nepal. Since the start of the VL elimination initiative in 2005, the number of cases of this deadly parasitic disease has decreased significantly in all three countries. Reported incidence rates now hover close to the target of less than 1/10,000 per year at (sub-)district level [1]. In this endgame period, identifying and reaching specific risk groups will be crucial to meet and sustain the elimination target.

One recurrent observation is that patients treated for VL in health services are predominantly male [2, 3]. This is not limited to the Indian subcontinent but was observed across different geographical locations, over different leishmanial species and disease spectra, including cutaneous forms of leishmaniasis [2, 4–6]. Two hypotheses have been formulated to explain this difference between both sexes, stressing either social differences between men and women (i.e., *gender* differences, relating to the specific behavior, activities, and roles of men and women in society) or biological differences (i.e., *sex* differences) [7]. Different behaviors may result in less exposure to infection for women or less access to care when sick. Several years ago, Ahluwalia *et al*. showed in Bangladesh that women suffered from higher VL case fatality rates, most likely due to gender discrimination in access to care [8]. A more recent study in India confirmed the low prioritization of women’s health as an important factor of delayed treatment seeking among females [9]. If this is also the case in India and Nepal, with a higher proportion of women remaining undiagnosed and underreported, this would be an essential issue to address.

An alternative hypothesis suggests that physiological differences in host-pathogen interactions and immune response after infection lie at the basis of the male predominance in VL. Hormones, including sex hormones, can modulate the immune system and influence the immune response to infection with various pathogens. Several animal studies support the hypothesis that this physiological difference also plays a role in leishmaniasis, and have described higher susceptibility to disease and worse disease outcomes after *Leishmania* infection in male animals [10–13]. However, there are, so far, no studies investigating the reasons for this male predominance in reported VL on the Indian subcontinent. By analyzing the sex ratio of VL outcomes in population-based studies conducted in the same region as for which health systems data are available, we explored the hypothesis that gender-based differences in health care-seeking behavior and access to care explain this gender imbalance in reported cases. In these longitudinal population-based studies, incident VL was systematically recorded over several years for all individuals residing within defined areas through door-to-door household surveys. We can, therefore, safely assume that the population data on male and female VL patients were captured with equal validity, and the impact of any potential difference in health care seeking behavior or access to care was minimal in these data. This provides a unique opportunity to test the nature versus nurture hypothesis to explain the male VL predominance.

## Methods

### Data included for analysis

We collected data from official health care reporting systems in India and Nepal. These data include all VL cases that were reported through the national health system (Nepal) or the public health services of the respective states in India, and are mainly based on passive case detection at primary care facilities. From India, all VL cases reported between January 2014 and July 2017 through the Kala Azar Management Information System (KAMIS) from the highly VL endemic states of Bihar and Jharkhand were included. For Nepal, all VL cases reported to the national registry from the Epidemiology and Disease Control Department between January 2014 and December 2017 were included for analysis.

Population-based data were obtained from two cohort studies; Kalanet and the Tropical Medicine Research Center (TMRC) Health and Demographic Surveillance System (HDSS). The Kalanet study was carried out between 2006 and 2009 in 26 VL endemic clusters in both India and Nepal, covering a total of 12,691 participants for three consecutive years. It included three consecutive (yearly) serological measurements with Direct Agglutination Test (DAT) together with an active tracing of any incident disease in quarterly household surveys. The second cohort used was the Muzaffarpur-TMRC HDSS, located in Muzaffarpur district, Bihar state, India. The HDSS was established in 2007, covering 50 adjacent villages with a population of over 90,000 people (‘old area’), to which a new (contiguous) area of 16 scattered villages (20,000 people) was added in 2012 (‘new area’). In 2009 an exhaustive serological survey was conducted in the 11 most endemic villages of the old area using the Direct Agglutination Test (DAT) and repeated the following year to establish the number of new asymptomatic infections through seroconversion. In 2012 this method was repeated for the 16 ‘new area’ villages. All participants of the population-based longitudinal studies were followed up every month through a network of community health workers and field teams for detecting incident VL; we can, therefore, assume the number of unreported VL patients to be extremely low.

### Statistical analyses

Data analysis was performed with Stata 14 [StataCorp, Texas, USA]. Age was categorized into five age groups; 0–14 years old, 15–29 years old, 30–44 years old, 45–59 years old, and ≥60 years old. These boundaries of age groups were set, taking into account the available sample size per age group. Routine health systems data were analyzed to calculate the male/female ratio among all VL cases reported per age group, together with a 95% Confidence Interval (CI), using logistic regression. Also, a χ^2^ test for trend was done to test for an age-dependent trend in the male/female ratio among VL cases. The cohort data from the Kalanet and TMRC study were analyzed as follows. The overall male/female ratio among all incident VL was calculated per age group, including 95% CI, using logistic regression, and a χ^2^ test for trend was done to look for an age-dependent trend in the male/female ratio among VL cases. Proportions of seropositive individuals at baseline, defined as a DAT titer ≥ 1:3,200, were calculated per sex and per age group. Seroconversion, defined as a baseline DAT titer < 1:3,200 in combination with a DAT titer ≥ 1:3,200 and at least two titer steps higher than the baseline titer at any time thereafter, was represented by the cumulative incidence per sex and age group. To be able to compare the data meaningfully across study sites, we assessed male sex as a potential risk factor for seroprevalence at baseline, seroconversion, VL incidence, VL incidence among seroconverters (Kalanet data only), and persistent seropositivity one year after seroconversion (Kalanet data only) by using Poisson regression controlling for age and study site. Results were expressed by risk ratios (including 95% CI) of males versus females.

### Ethical approval

All data analyzed were anonymized. Ethical approval was obtained from the Banaras Hindu University, Varanasi, India.

## Results

### Health systems data

**Fig 1:**
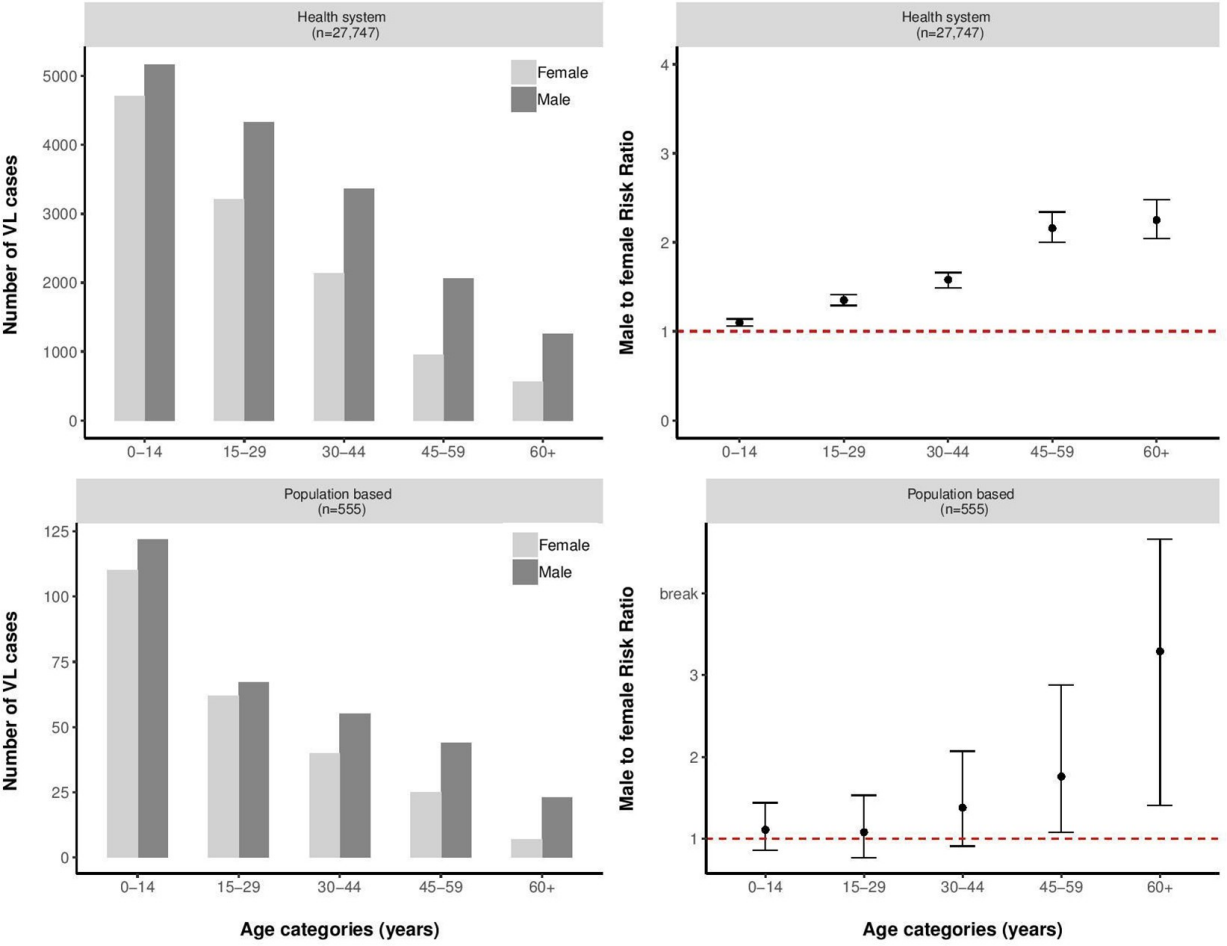
Absolute number of patients per sex and age group, and male to female ratio per age group of patients with visceral leishmaniasis reported through the health systems (Bihar and Jharkhand, India (2014–2017; n = 26,845), and Nepal (2014–2017; n = 902)) and through population-based studies (Kalanet (2006–2009; n = 109) and TMRC (2007(2012 for ‘new area’)– 2015; n = 555))).

A total of 27,747 VL cases reported through the routine health system of India and Nepal were included; 26,845 from India (21,215 from Bihar and 5,630 from Jharkhand State) and 902 from Nepal. The median age of all VL patients was 21 years (IQR 11 to 35), with the median age in Nepal being significantly higher (28 years; IQR 16 to 43) than in India (21 years; IQR 11 to 35) (p-value <0.001). The median age of VL patients was higher in males (24 years; IQR 12 to 40) than in females (18 years; IQR 10 to 32) (p-value <0.001). Overall, 16,182 (58.32%) VL patients were male and 11,565 (41.68%) were female, corresponding to a male/female ratio of 1.40 (95% CI 1.37–1.43). Study-site specific male/female ratios were 1.37 (95% CI 1.34–1.41) in Bihar (India), 1.44 (95% CI 1.37–1.52) in Jharkhand (India), and 1.78 (95% CI 1.54–2.01) in Nepal, showing a significantly higher male/female ratio in Nepal than in India (p-value = 0.001). Fig 1 graphically represents the absolute number of VL cases per sex as well as the male/female ratio of VL cases per age category in all health systems data combined. The male/female ratio of VL cases in the combined health systems data increased from 1.10 (95% CI 1.06–1.14) in the youngest age group to 1.35 (95% CI 1.29–1.41), 1.58 (95% CI 1.49–1.66), 2.16 (95% CI 2.04–2.48), and 2.25 (95% CI 2.04–2.48) with increasing age groups (p-value for χ^2^ for trend < 0.001). Apart from a significantly higher male/female ratio in the youngest age group in Nepal when compared to that in India (1.64 versus 1.09; p = 0.006), a similar age-specific pattern was observed across all study sites. Details on patient characteristics and male/female ratios of VL cases per study site are presented in S1 Table.

### Longitudinal cohort studies

A total of 119,172 participants were followed up through the population-based longitudinal studies for the assessment of incident VL; 105,886 through the TMRC study and 13,286 through the Kalanet study (7,950 from India and 5,336 from Nepal). Overall, 50% of the included population were male, and the median age was 20 years (IQR 10–38), without significant differences among the study sites. A total of 555 new cases of VL were observed; 109 during Kalanet (82 in India, 27 in Nepal) and 446 during the TMRC study (325 in the ‘old area’ and 121 in the ‘new area’). Overall, the median age of VL patients identified through the cohort studies was 18 years (IQR 9–39). The median age of VL patients identified was significantly different among the specific study-sites; 10 years in Kalanet India (IQR 7–27), 35 in Kalanet Nepal (IQR 14–42) and 19 years in TMRC (IQR 9–39) (p-value <0.001). Median age in male VL patients was significantly higher than in females; 19 years (IQR 9–42) for males versus 16 years (IQR 8–33) for females (p-value = 0.001). Overall, 311 out of 555 VL patients (56.0%) were male and 244 (44.0%) were female, corresponding to a male/female ratio of 1.27 (95% CI 1.08–1.51). The small numbers of incident VL cases in the different study-sites did not allow for a meaningful comparison of male/female ratios between study groups. Fig 1 graphically represents the absolute number of VL cases per sex as well as the male/female ratio of VL cases per age category in all cohort study sites combined. The male/female ratio of VL cases in the combined cohort data increased from 1.11 (95% CI 0.86–1.44) in the youngest age group to 1.08 (95% CI 0.77–1.53), 1.38 (95% CI 0.91–2.07), 1.76 (95% CI 1.08–2.88), and 3.29 (95% CI 1.41–7.66) with increasing age group, though this trend was not significant (p-value for χ^2^ for trend = 0.072). Details on patient characteristics and male/female ratios of VL cases per study site are presented in S2 Table.

**Fig 2:**
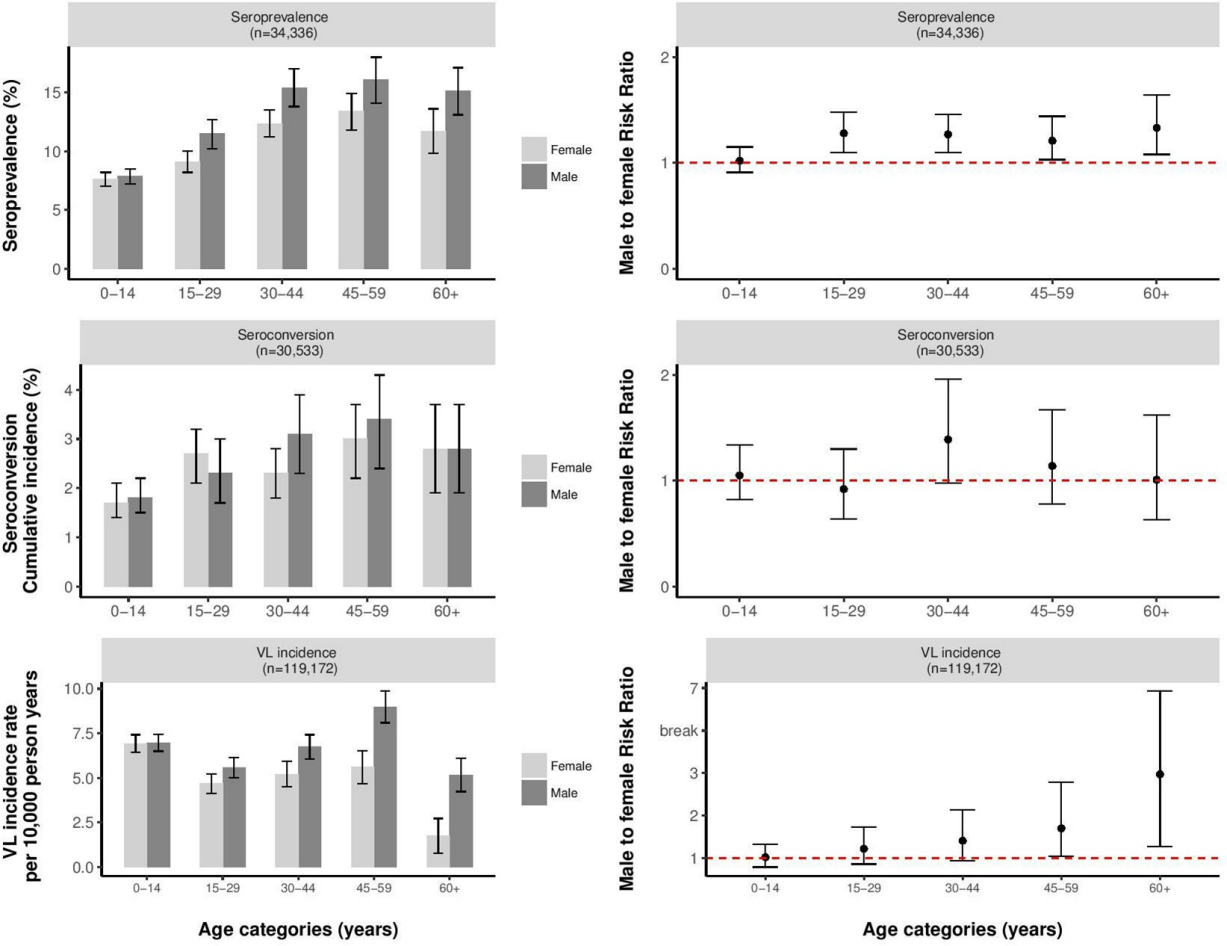
Seroprevalence (Direct Agglutination Test titer ≥ 1:3,200), seroconversion rate as a cumulative incidence and visceral leishmaniasis incidence rate per 10,000 person years, are presented on the left panel of the figure per age group and including 95% confidence intervals. On the right panel of the figure the respective relative risks for males compared to females are represented by age group, including 95% confidence intervals. Seroprevalence figures are based on population-based longitudinal data of Kalanet (2006–2009; n = 13,286) and TMRC (2009–2010 (2012–2013 for ‘new area’); n = 21,050). Seroconversion figures are based on population longitudinal data, including individuals for whom at least two serological results were available from Kalanet (2006–2009; n = 12,537) and TMRC (2009–2010 (2012–2013 for ‘new area’); n = 17,996). VL incidence rates are based on population-based longitudinal data of Kalanet (2006–2009; n = 13,286) and TMRC (2007 (2012 for ‘new area’)– 2015; n = 105,886).

The overall VL incidence rate for females in the cohort studies was 5.39/10,000 person-years (95% CI 5.10–5.68) as opposed to 6.61/10,000 person-years for males (95% CI 6.32–6.89). Age group and sex-specific incidence rates are listed in S3 Table. After controlling for study site and age category, the relative risk for males compared to females to develop VL was 1.27 (95% CI 1.08–1.51). Age-specific relative risk for males was 1.02 (95% CI 0.79–1.32) in the youngest age group, 1.22 (95% CI 0.86–1.73) in the age group of 15 to 29 years, 1.41 (95% CI 0.94–2.13) in the age group of 30 to 44 years, 1.70 (95% CI 1.04–2.78) in the age group of 45 to 59 years, and 2.97 (95% CI 1.27–6.93) in the eldest age group. Fig 2 graphically represents the VL incidence rates per sex and per age group, as well as the risk ratio of males versus females per age group for developing VL.

Serological data were available for a total of 34,336 participants; 7,950 in Kalanet India, 5,336 in Kalanet Nepal, and 21,050 in TMRC. Overall, 46.5% of the participants were male, and the median age of participants was 18 years (IQR 8–38). Median age was similar among different study sites (18 years in Kalanet India and TMRC, 22 years in Kalanet Nepal). Exact sex- and age-specific numbers can be found in S4 Table. Overall, 3,572 out of 34,336 participants (10.4%; 95% CI 10.1% - 10.7%) were seropositive at baseline; 1,821 from TMRC area, 1,280 from Kalanet India, and 471 from Kalanet Nepal. The overall seroprevalence was 11.1% (95% CI 10.6% - 11.6%) in males and 9.8% (95% CI 9.3% - 10.2%) in females (p-value < 0.001), increasing with age from 7.6% (95% CI 7.0% - 8.2%) to 11.7% (95% CI 9.8%– 13.6%) in females and from 7.9% (95% CI 7.2% - 8.5%) to 15.1% (95% CI 13.1% - 17.1%) in males. Exact numbers on seropositive individuals per sex and age group are listed in S4 Table. Age and sex-specific seroprevalence rates are listed in S3 Table. After controlling for study site and age category, the overall risk ratio of males versus females to be seropositive at baseline was RR 1.19 (95% CI 1.11–1.27), with age-specific risk ratios of 1.02 (95% CI 0.91–1.15) in the youngest age group, 1.28 (95% CI 1.10–1.48) in the age group of 15 to 29 years, 1.27 (95% CI 1.10–1.46) in the age group of 30 to 44 years, 1.21 (95% CI 1.03–1.44) in the age group of 45 to 59 years, and 1.33 (95% CI 1.08–1.64) in the eldest age group. Fig 2 graphically represents the proportion of individuals seropositive at baseline per sex and per age group, as well as the risk ratio of males versus females per age group to be seropositive at baseline.

For 30,533 participants at least two serological results were available. Among these participants, a total of 701 seroconversions were registered; 383 through Kalanet (290 in India, 93 in Nepal) and 318 through TMRC (180 in the ‘old area’, 138 in the ‘new area’). Overall, 44.9% of seroconverters were male, and the median age of seroconverting was 25 years (IQR 10–45). The overall cumulative incidence of seroconversion was 2.3% (95% CI 2.1% - 2.5%) and showed no difference between males and females; 2.3% (95% CI 2.0% - 2.5%) for females and 2.3% (95% CI 2.1% - 2.6%) for males, increasing with age from 1.7% (95% CI 1.4% - 2.1%) to 2.8% (95% CI 1.9% - 3.7%) in females and from 1.8% (95% CI 1.5% - 2.2%) to 2.8% (95% CI 1.9% - 3.7%) in males. Age and sex-specific seroconversion rates are listed in S3 Table. After controlling for study site and age category, the overall relative risk for males versus females for seroconversion was 1.09 (95% CI 0.93–1.26), with an age-specific relative risk for males of 1.05 (95% CI 0.82–1.34) in the youngest age group, 0.92 (95% CI 0.64–1.30) in the age group of 15 to 29 years, 1.39 (95% CI 0.98–1.96) in the age group of 30 to 44 years, 1.14 (95% CI 0.78–1.67) in the age group of 45 to 59 years, and 1.01 (95% CI 0.63–1.62) in the eldest age group. Fig 2 graphically represents the seroconversion rates per sex and per age group, as well as the risk ratio of males versus females per age group for seroconversion.

In additional analyses on the three serological data points available in the Kalanet data, males over the age of 15 years were found to have a higher risk of developing VL in the first year after seroconversion (RR 1.96; 95% CI 0.82–4.71) and after being seropositive at baseline (RR 1.91; 95% CI 0.70–5.18). Additionally, they were found to have a higher chance of remaining seropositive one year after seroconversion (RR 1.35; 95% CI 0.75–2.44).

## Discussion

In this large multi-country study in the Indian subcontinent, male VL cases were significantly more frequently reported by the health services than female VL cases, with a male to female sex ratio of 1.40 (95% CI 1.37–1.43). This male predominance was also present in population-based cohort studies, in which the population at risk was actively screened for VL on a door-to-door basis at regular intervals, revealing a male to female risk ratio for incident VL of 1.27 (95% CI 1.08–1.51). This suggests that the sex imbalance apparent in reported VL is not primarily due to socio-cultural differences in access to care and health-seeking behavior, but that biological factors play a role as well.

In addition, the population-based cohort studies showed that males had a higher chance of being seropositive in DAT at baseline (RR 1.19; 95% CI 1.11–1.27), but only after the age of 15 years, while no difference in seroconversion rates between both sexes was observed at the DAT cut-off we used as the primary endpoint to define incident infection. However, after seroconversion, males over the age of 15 years were at higher risk of developing the disease than females, though not significantly so (RR 1.96; 95% CI 0.82–4.71). Males were also found to be at higher risk than females to develop VL disease if being seropositive at baseline (1.91; 95% CI 0.70–5.18), and were more likely to remain seropositive one year after seroconversion (RR 1.35; 95% CI 0.75–2.44), though the difference was not significant. Interestingly, for children under the age of 15 years, there was no increased risk for males to develop VL or be seropositive at baseline (IRR 1.02 (95% CI 0.79–1.32) and RR 1.02 (95% CI 0.91–1.15) respectively).

The main limitations of this study were the relatively small number of VL cases occurring in the cohort studies, limiting the power of the study when analyzing the data per country (especially for Nepal) or in smaller age groups. In addition, the data obtained through the routine health systems from India and Nepal were collected in more recent years (2014–2017) than the data obtained through the cohort studies (2007–2015), which may have influenced the results. However, an analysis on VL case data from one kala-azar specialized hospital in Bihar, India (Kala-Azar Medical Research Center) between 2002 and 2017 revealed no changing trend in the sex ratio of the cases over time (data presented in S5 Table), so we do not think that this would have a significant impact on the validity of the findings. Seroprevalence and seroconversion were used (measured by DAT with cut-off ≥ 1:3,200) as proxy measures for existing infection and new infection respectively, and as these are not an exact measurement of both parameters, results should be interpreted with care. In addition, there is no validated and universally accepted cut-off value for the Direct Agglutination Test to define leishmanial infection. While a value of ≥ 1:3,200 is the more commonly used cut-off to diagnose clinical disease, many serological studies have used the lower cut-off of ≥ 1:1,600 to define infection, aiming for higher sensitivity. As lower DAT cut-off titers are less specific, and higher titers have been shown to have prognostic value for identifying those among the infected who will progress to disease [14], we decided to use a DAT cut-off of ≥ 1:3,200 to define infection in this paper. A secondary analysis for a DAT cut-off value of ≥ 1:1,600 was added in the Supplementary materials (S1 Fig, S6 Table).

We recognize that the youngest age group of 0–14 years is a heterogenous group, and therefore interpreting and generalizing the findings in this group should be done with care. Infants have been shown to go through a “mini-puberty” with a peak of sex hormones in their first year of life which may influence a sex-biased susceptibility to disease in this subgroup [15]. As children under the age of two years were excluded from the serological surveys, and VL cases in this subgroup were too few to make any meaningful comparisons between both sexes, this subgroup could not be analyzed separately. In addition, some teenagers might have gone through puberty before the age of 15, and might therefore have biological features more similar to those of young adults.

Our data are in agreement with several other studies and reports describing a male predominance in VL cases, on the Asian, African, and South American continents [2, 3, 5, 7, 16, 17]. Most often, these studies were based on health-facility data, but other longitudinal population-based surveys have confirmed these findings [5, 18]. Data available in the literature on the risk of exposure to infection of both sexes are contradictory [3, 7, 19], and most often are based on a positive serological (DAT or rK39) or cellular immunity (leishmanin skin) test. Any conclusions on exposure based on prevalence data, however, should be interpreted with care, as duration of a measurable immune response after exposure to *Leishmania* parasites may already be gender-biased, as was suggested—though not statistically significantly so—in this study by the fact that males were found to have a higher chance of remaining seropositive one year after seroconversion (RR 1.35; 95% CI 0.75–2.44). In our study, seroprevalence was higher in males than in females, which as such could be associated to gender-related differences in exposure risk. If that were the case, however, we would expect higher seroconversion rates in males, which was not illustrated by our data. Seroconversion as a proxy for new infection should on the other hand also be interpreted with caution, as factors other than exposure to infection could influence the risk of seroconversion. In line with previous literature, our study suggested age to impact the risk of seroconversion for leishmania [17], but other risk factors such as nutritional status and overall health have been suggested to alter the risk of seroconversion for other infectious agents [17, 20, 21]. While the overall risk of seroconversion was not different between males and females, we did find age-group specific differences, with higher risk of seroconversion in males aged 30–44 years, while in the age group of 15–29 year olds the opposite was true, although both results were not statistically significant. Whether or not these differences are the result of age-specific gender-related differences in exposure to infection the presence of different sex hormones in both sexes, or other unknown factors, is not clear.

We do not claim that all the excess VL in male can be explained by biological factors in this context. In Bangladesh, where women were found to be ill longer before receiving treatment, women’s lower status within the household and treatment prioritization of children and income earners before women were among the main reasons for differences in health care-seeking behavior [8]. Burza *et al*. also found women in India to be at risk of presenting at the hospital in a later stage of the disease [2]. However, our data show that the socio-cultural determinant of women’s discrimination cannot be the main explanation for the male predominance in VL in this context, and suggest the need to disentangle social from biological determinants on the Indian subcontinent for coherent and effective targeted elimination efforts to be developed.

Our data strongly suggest a role for sex-related biological factors in the pathogenesis of leishmaniasis. Together with a higher risk of developing VL, males over the age of 15 years show a higher seroprevalence than females, and are more at risk of developing symptoms once infected. What sex-related differences are responsible for this, however, remains unclear.

The role of sex hormones in influencing the immune system and altering pathogenesis of leishmaniasis has been established. Several animal studies, where males and females were equally exposed to infection, have greatly contributed to this understanding. In general, male animals seem to have a higher susceptibility and disease severity, as assayed by the number and size of lesions as well as by the parasite burden [10–13]. In some models, these differences were neutralized by hormonal interventions; testosterone-treated females showed aggravated disease signs, while males after orchidectomy or before the age of puberty seemed to be relatively protected [10, 12]. Testosterone was also shown to increase the uptake of *L*. *donovani* parasites by macrophages, and increase infection rates and infection levels of these cells in vitro [22], suggesting a direct effect of this hormone. On the other hand, in some models exogenous estrogen administration was shown to increase leishmanicidal activity in macrophages in both male and female mice, while this effect was only present in female mice in another model [23, 24]. The role of sex-hormones seems in line with the findings in this and other studies that sex-differences are absent in children before the age of puberty [5, 7, 13, 25, 26]. However, the sex-bias in this study increased with increasing age of the participants, which cannot be explained by mere sex hormone levels, as these decrease at a later age. This shows that other factors must also play a role. Recent studies have shown that epigenetics and gene expression in various cell types, including immune cells, can be sex-dependent as well [27]. Whether or to what extent each of these mechanisms plays a role in the pathogenesis of leishmaniasis needs to be established further.

The severity and outcome of disease seems also to be sex-dependent, with male sex being suggested as a risk factor for death or relapse after treatment–once again especially after the age of puberty [1, 25, 28–33]. Unfortunately, no information on outcome of the disease was available for our study. Important to note is that these sex-related differences seem to be dependent on the leishmania species, the host species, and the affected tissue [34, 35]. While all data on *L*. *donovani* show the incidence of VL in humans to be higher in males than in females, for *L*. *tropica* the opposite has been observed in more than one study [36, 37]. This shows that more factors—largely unknown- define the final response of an affected host to this parasite.

The potential role for sex-related factors in the pathogenesis of leishmaniasis could open perspectives for new therapeutic approaches. Tamoxifen, an antagonist of the estrogen receptor, has already been suggested as a new oral drug candidate for the treatment of *L*. *major* and *L*. *amazonensis* [38–40]. Moreover, as it is highly likely that the mechanisms through which sex-related differences influence the immune system are similar in several infectious diseases, this might have implications for drug development in other infectious diseases as well.

Summarizing, this study shows that differences in health care-seeking behavior and access to health care between men and women are not the main reason for the observed male predominance of VL cases on the Indian subcontinent. We suggest that mainly sex-related biological factors are responsible for this difference; the specific factors involved remain to be established.

## Supporting information

S1 Fig Seroprevalence (Direct Agglutination Test titer ≥ 1:1,600), seroconversion rate as a cumulative incidence and visceral leishmaniasis incidence rate per 10,000 person years, are presented on the left panel of the figure per age group and including 95% confidence intervals. On the right panel of the figure the respective relative risks for males compared to females are represented by age group, including 95% confidence intervals. Seroprevalence figures are based on population-based longitudinal data of Kalanet (2006–2009; n = 13,286) and TMRC (2009–2010 (2012–2013 for ‘new area’); n = 21,050). Seroconversion figures are based on population longitudinal data, including individuals for whom at least two serological results were available from Kalanet (2006–2009; n = 12,537) and TMRC (2009–2010 (2012–2013 for ‘new area’); n = 17,996). VL incidence rates are based on population-based longitudinal data of Kalanet (2006–2009; n = 13,286) and TMRC (2007 (2012 for ‘new area’)– 2015; n = 105,886).
(TIF)

S1 Table Characteristics of patients with visceral leishmaniasis reported through the health systems of Bihar, India (2014–2017; n = 21,215), Jharkhand, India (2014–2017; n = 5,630) and Nepal (2014–2017; n = 902). (DOCX)

S2 Table Characteristics of patients with visceral leishmaniasis identified through population-based longitudinal studies of Kalanet (2009–2009; n = 109) and TMRC (2007 (2012 for ‘new area’)– 2015; n = 555). (DOCX)

S3 Table Seroprevalence (%), seroconversion (%) and incidence rate per 10,000 person years per sex and per age group as observed through population-based longitudinal studies (Kalanet (2006–2009) and TMRC (2009–2010 (2012–2013 for ‘new area’) for serological data and 2007 (2012 for ‘new area’)– 2015 for VL incidence data)). Direct Agglutination Test cut-off titer of ≥ 1:3,200 was used to define seropositivity.
(DOCX)

S4 Table Characteristics of individuals for whom a serological result was available through population-based longitudinal studies (Kalanet (2006–2009; n = 13,286) and TMRC (2009–2010 (2012–2013 for ‘new area’); n = 21,050). 30,533 participants–for whom also a second serological result was available—were included to evaluate seroconversion (Kalanet (2006–2009; n = 12,537) and TMRC (2009–2010 (2012–2013 for ‘new area’); n = 17,996). Direct Agglutination Test cut-off titer of ≥ 1:3,200 was used to define seropositivity.
(DOCX)

S5 Table Absolute numbers of patients diagnosed with visceral leishmaniasis in Kala-Azar Medical Research Center, Muzaffarpur, Bihar, India between 2002–2017. No trend over time of the male to female ratio was found (z-score of non-parametric test for trend = 0.25).
(DOCX)

S6 Table Risk ratio of males versus females for seroprevalence at baseline and seroconversion as observed through population-based longitudinal studies (Kalanet (2006–2009; n = 13,286) and TMRC (2009–2010 (2012–2013 for ‘new area’); n = 21,050). 30,533 participants–for whom also a second serological result was available—were included to evaluate seroconversion (Kalanet (2006–2009; n = 12,537) and TMRC (2009–2010 (2012–2013 for ‘new area’); n = 17,996). A Direct Agglutination Test cut-off of ≥ 1:1,600 was used to define seropositivity. Direct Agglutination Test cut-off titer of ≥ 1:1,600 was used to define seropositivity.
(DOCX)
