# Supplementary figures and images for: Male predominance in reported Visceral Leishmaniasis cases: Nature or nurture? A comparison of population-based with health facility-reported data
Source: PLoS Negl Trop Dis. 2020 Jan 29;14(1):e0007995. doi: 10.1371/journal.pntd.0007995 (PMC7010295; doi:10.1371/journal.pntd.0007995)

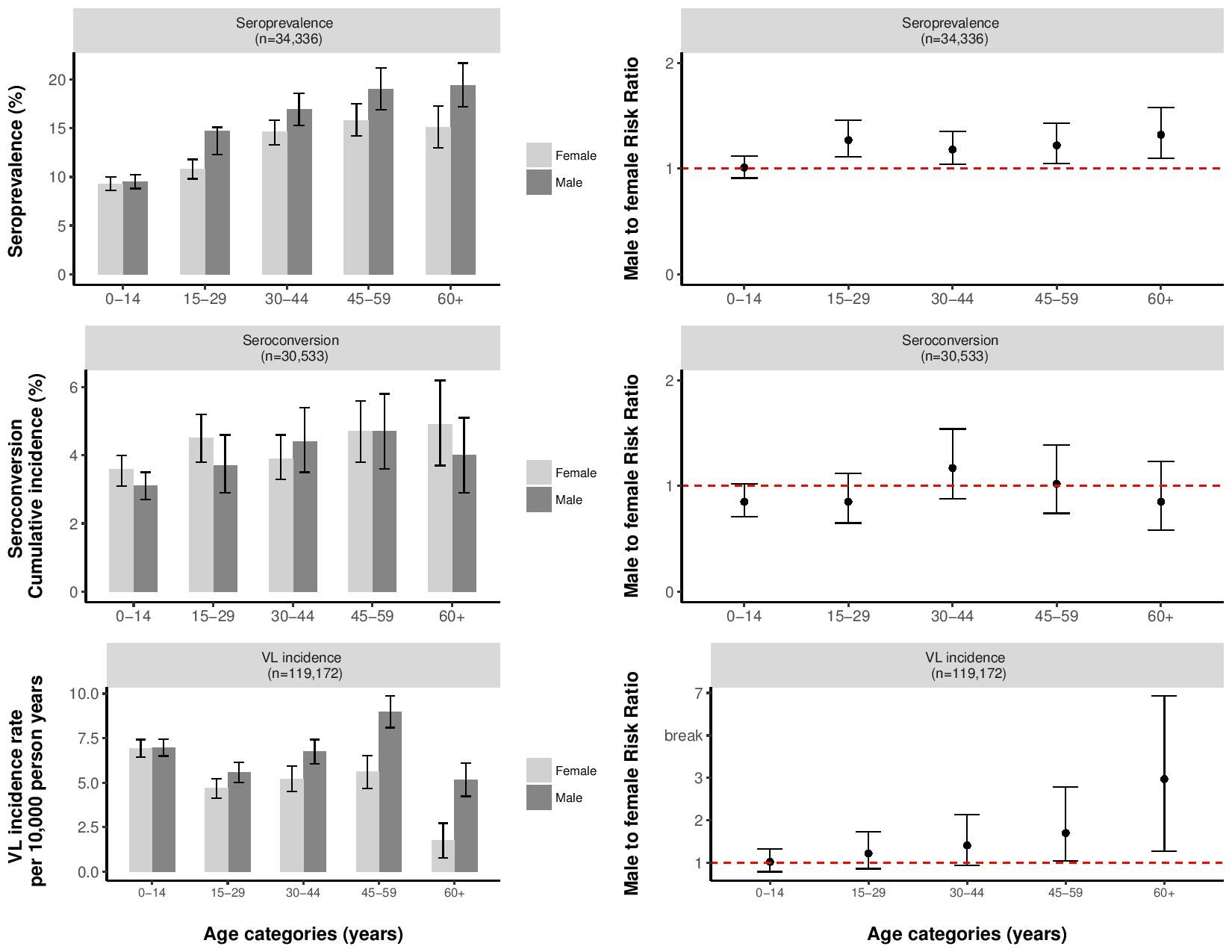

Supplement: S1 Fig — On the right panel of the figure the respective relative risks for males compared to females are represented by age group, including 95% confidence intervals. Seroprevalence figures are based on population-based longitudinal data of Kalanet (2006–2009; n = 13,286) and TMRC (2009–2010 (2012–2013 for ‘new area’); n = 21,050). Seroconversion figures are based on population longitudinal data, including individuals for whom at least two serological results were available from Kalanet (2006–2009; n = 12,537) and TMRC (2009–2010 (2012–2013 for ‘new area’); n = 17,996). VL incidence rates are based on population-based longitudinal data of Kalanet (2006–2009; n = 13,286) and TMRC (2007 (2012 for ‘new area’)– 2015; n = 105,886). (TIF) [file pntd.0007995.s001.tif]
