# Supplementary material for: Male predominance in reported Visceral Leishmaniasis cases: Nature or nurture? A comparison of population-based with health facility-reported data
Source: PLoS Negl Trop Dis. 2020 Jan 29;14(1):e0007995. doi: 10.1371/journal.pntd.0007995 (PMC7010295; doi:10.1371/journal.pntd.0007995)
Supplement: S1 Table — (DOCX) [file pntd.0007995.s002.docx]

**S1 Table : Patient characteristics of patients with visceral leishmaniasis reported through the health systems of Bihar, India (2014-2017 ; n = 21,215), Jharkhand, India (2014 – 2017 ; n = 5,630) and Nepal (2014 – 2017 ; n = 902).**

|  |  |  |  |  |  |  |  |  |  |
| --- | --- | --- | --- | --- | --- | --- | --- | --- | --- |
| **Patient characteristics** | | **Bihar (India)**  **(n = 21,215)** | | **Jharkhand (India)**  **(n = 5,630)** | | **Nepal**  **(n = 902)** | | **Overall**  **(n = 27,747)** | |
| **Sex** | |  |  |  |  |  |  |  |  |
|  | Male | 12,280 (57.9%) | | 3,327 (59.1%) | | 575 (63.7%) | | 16,182 (58.3%) | |
|  | Female | 8,935 (42.1%) | | 2,303 (40.9%) | | 327 (36.3%) | | 11,565 (41.7%) | |
| **Median age (years (IQR))** | | 21 (10 to 37) | | 22 (12 to 35) | | 28 (16 to 43) | | 21 (11 to 35) | |
| **Age group** |  | **Male/female**  **(n)** | **M/F ratio**  **(95% CI)** | **Male/female**  **(n)** | **M/F ratio**  **(95% CI)** | **Male/female**  **(n)** | **M/F ratio**  **(95% CI)** | **Male/female**  **(n)** | **M/F ratio**  **(95% CI)** |
|  | 0-14 | 4,072/3,775 | **1.08** (1.03 - 1.13) | 971/856 | **1.13** (1.03 - 1.24) | 128/78 | **1.64** (1.24 - 2.17) | 5,171/4,709 | **1.10** (1.06 - 1.14) |
|  | 15-29 | 3,053/2,300 | **1.33** (1.26 - 1.40) | 1,123/800 | **1.40** (1.28 - 1.54) | 155/111 | **1.40** (1.09 - 1.78) | 4,331/3,211 | **1.35** (1.29 - 1.41) |
|  | 30-44 | 2,481/1,629 | **1.52** (1.43 - 1.62) | 738/433 | **1.70** (1.51 - 1.92) | 142/71 | **2.00** (1.50 - 2.66) | 3,361/2,133 | **1.58** (1.49 - 1.66) |
|  | 45-59 | 1,576/738 | **2.14** (1.96 - 2.33) | 377/165 | **2.28** (1.90 - 2.74) | 107/49 | **2.18** (1.56 - 3.06) | 2,060/952 | **2.16** (2.00 - 2.34) |
|  | 60+ | 1,098/493 | **2.23** (2.00 - 2.48) | 118/49 | **2.41** (1.73 - 3.36) | 43/18 | **2.39** (1.38 - 4.14) | 1,259/560 | **2.25** (2.04 - 2.48) |
| **Total** | | 12,280/8,935 | **1.37** (1.34 - 1.41) | 3,327/2,303 | **1.44** (1.37 - 1.52) | 575/327 | **1.76** (1.54 - 2.01) | 16,182/11,565 | **1.40** (1.37 - 1.43) |
|  |  |  |  |  |  |  |  |  |  |
