# Supplementary material for: Male predominance in reported Visceral Leishmaniasis cases: Nature or nurture? A comparison of population-based with health facility-reported data
Source: PLoS Negl Trop Dis. 2020 Jan 29;14(1):e0007995. doi: 10.1371/journal.pntd.0007995 (PMC7010295; doi:10.1371/journal.pntd.0007995)
Supplement: S2 Table — (DOCX) [file pntd.0007995.s003.docx]

**S2 Table : Patient characteristics of patients with visceral leishmaniasis identified through population-based longitudinal studies of Kalanet (2009-2009 ; n = 109) and TMRC (2007 (2012 for new area) – 2015 ; n = 555).**

| **Patient characteristics** | | **Kalanet India**  **(n = 82)** | | **Kalanet Nepal**  **(n = 27)** | | **TMRC**  **(n = 446)** | | **Overall**  **(n = 555)** | |
| --- | --- | --- | --- | --- | --- | --- | --- | --- | --- |
| **Sex** | |  |  |  |  |  |  |  |  |
|  | Male | 44 (53.7%) | | 13 (48.1%) | | 254 (57.0%) | | 311 (56.0%) | |
|  | Female | 38 (46.3%) | | 14 (51.9%) | | 192 (43.0%) | | 244 (44.0%) | |
| **Median age (years (IQR))** | | 10 (7 - 27) | | 35 (14 - 42) | | 19 (9 - 39) |  | 18 (9 - 39) | |
| **Age groups** | | **Male/female (n)** | **M/F ratio**  **(95% CI)** | **Male/female (n)** | **M/F ratio**  **(95% CI)** | **Male/female (n)** | **M/F ratio**  **(95% CI)** | **Male/female (n)** | **M/F ratio**  **(95% CI)** |
|  | 0-14 | 27/24 | 1.13 (0.65 - 1.95) | 1/7 | 0.14 (0.02 - 1.16) | 94/79 | 1.19 (0.88 - 1.60) | 122/110 | 1.11 (0.86 - 1.44) |
|  | 15-29 | 5/6 | 0.83 (0.25 - 2.73) | 4/0 | NA | 58/56 | 1.04 (0.72 - 1.50) | 67/62 | 1.08 (0.77 - 1.53) |
|  | 30-44 | 5/3 | 1.67 (0.40 - 6.97) | 4/5 | 0.80 (NA - NA) | 46/32 | 1.44 (0.92 - 2.26) | 55/40 | 1.38 (0.91 - 2.07) |
|  | 45-59 | 4/4 | 1.00 (0.25 - 4.00) | 2/1 | 2.00 (NA - NA) | 38/20 | 1.90 (1.11 - 3.27) | 44/25 | 1.76 (1.08 - 2.88) |
|  | 60+ | 3/1 | 3.00 (0.31 - 28.84) | 2/1 | 2.00 (NA - NA) | 18/5 | 3.60 (1.34 - 9.70) | 23/7 | 3.29 (1.41 - 7.66) |
| **Total** |  | 44/38 | **1.16 (0.75 - 1.79)** | 13/14 | **0.93 (NA - NA)** | 254/192 | **1.32 (1.10 - 1.60)** | 311/244 | **1.27 (1.08 - 1.51)** |
|  |  |  |  |  |  |  |  |  |  |
