# Supplementary material for: Male predominance in reported Visceral Leishmaniasis cases: Nature or nurture? A comparison of population-based with health facility-reported data
Source: PLoS Negl Trop Dis. 2020 Jan 29;14(1):e0007995. doi: 10.1371/journal.pntd.0007995 (PMC7010295; doi:10.1371/journal.pntd.0007995)
Supplement: S3 Table — Direct Agglutination Test cut-off titer of ≥ 1:3,200 was used to define seropositivity. (DOCX) [file pntd.0007995.s004.docx]

**S3 Table : Seroprevalence (%), seroconversion (%) and incidence rate per 10,000 person years per sex and per age group as observed through population-based longitudinal studies (Kalanet (2006-2009) and TMRC (2009-2010 (2012-2013 for new area) for serological data and 2007 (2012 for new area) – 2015 for VL incidence data)). Direct Agglutination Test cut-off titer of ≥ 1:3,200 was used to define seropositivity.**

|  |  |  |  |  |  |  |
| --- | --- | --- | --- | --- | --- | --- |
|  | **Seroprevalence (%)**  **(95% CI)**  **(n = 34,336)** | | **Cumulative Incidence of seroconversion (%)(95% CI)**  **(n = 30,533)** | | **Incidence rate/10,000 person years**  **(95% CI)**  **(n = 119,172)** | |
| **Age group** | **Female** | **Male** | **Female** | **Male** | **Female (CI)** | **Male (CI)** |
| 0-14 | **7.6** (7.0 - 8.2) | **7.9** (7.2 - 8.5) | **1.7** (1.4 - 2.1) | **1.8** (1.5 - 2.2) | **6.9** (6.4 - 7.4) | **7.0** (6.5 - 7.4) |
| 15-29 | **9.1** (8.2 - 10.0) | **11.5** (10.2 - 12.7) | **2.7** (2.1 - 3.2) | **2.3** (1.7 - 3.0) | **4.7** (4.1 - 5.2) | **5.6** (5.0 - 6.1) |
| 30-44 | **12.3** (11.2 - 13.5) | **15.4** (13.8 - 17.0) | **2.3** (1.8 - 2.8) | **3.1** (2.3 - 3.9) | **5.2** (4.5 - 5.9) | **6.7** (6.1 - 7.4) |
| 45-59 | **13.4** (11.8 - 14.9) | **16.1** (14.1 - 18.0) | **3.0** (2.2 - 3.7) | **3.4** (2.4 - 4.3) | **5.6** (4.7 - 6.5) | **9.0** (8.1 - 9.9) |
| 60+ | **11.7** (9.8 - 13.6) | **15.1** (13.1 - 17.1) | **2.8** (1.9 - 3.7) | **2.8** (1.9 - 3.7) | **1.8** (0.8 - 2.7) | **5.2** (4.2 - 6.1) |
| **Total** | **9.8** (9.3 - 10.2) | **11.1** (10.6 - 11.6) | **2.3** (2.0 - 2.5) | **2.3** (2.1 - 2.6) | **5.4** (5.1 - 5.7) | **6.6** (6.3 - 6.9) |
|  |  |  |  |  |  |  |
