# Supplementary material for: Male predominance in reported Visceral Leishmaniasis cases: Nature or nurture? A comparison of population-based with health facility-reported data
Source: PLoS Negl Trop Dis. 2020 Jan 29;14(1):e0007995. doi: 10.1371/journal.pntd.0007995 (PMC7010295; doi:10.1371/journal.pntd.0007995)
Supplement: S4 Table — 30,533 participants–for whom also a second serological result was available—were included to evaluate seroconversion (Kalanet (2006–2009; n = 12,537) and TMRC (2009–2010 (2012–2013 for ‘new area’); n = 17,996). Direct Agglutination Test cut-off titer of ≥ 1:3,200 was used to define seropositivity. (DOCX) [file pntd.0007995.s005.docx]

**S4 Table : Participant characteristics of individuals for whom a serological result was available through population-based longitudinal studies (Kalanet (2006-2009 ; n = 13,286) and TMRC (2009-2010 (2012-2013 for new area) ; n = 21,050). 30,533 participants – for whom also a second serological result was available - were included to evaluate seroconversion (Kalanet (2006-2009 ; n = 12,537) and TMRC (2009-2010 (2012-2013 for new area) ; n = 17,996). Direct Agglutination Test cut-off titer of ≥ 1:3,200 was used to define seropositivity.**

|  |  |  |  |  |
| --- | --- | --- | --- | --- |
|  |  | **All participants** | **Seropositives at baseline** | **Seroconvertors** |
| **Sex** | |  |  |  |
|  | Male | 15,952 (46.5%) | 1,800 (50.4%) | 315 (44.9%) |
|  | Female | 18,384 (53.5%) | 1,772 (49.6%) | 386 (55.1%) |
| **Median age (years (IQR))** | | 18 (8 - 38) | 28 (11 - 45) | 25 (10 - 45) |
| **Age groups (years)** | | **Total (male/female)** | **Total (male/female)** | **Total (male/female)** |
|  | 0-14 | 14,965 (7,679/7,286) | 1158 (604/554) | 248 (130/118) |
|  | 15-29 | 7,010 (2,840/4,170) | 704 (326/378) | 143 (46/97) |
|  | 30-44 | 5,868 (2,349/3,519) | 795 (361/434) | 133 (55/78) |
|  | 45-59 | 3,765 (1,638/2,127) | 547 (263/284) | 108 (48/60) |
|  | 60+ | 2,728 (1,446/1,282) | 368 (218/150) | 69 (36/33) |
| **Total** | | **34,336 (15,952/18,384)** | **3572 (1,772/1,800)** | **701 (315/386)** |
|  |  |  |  |  |
