# Supplementary material for: Male predominance in reported Visceral Leishmaniasis cases: Nature or nurture? A comparison of population-based with health facility-reported data
Source: PLoS Negl Trop Dis. 2020 Jan 29;14(1):e0007995. doi: 10.1371/journal.pntd.0007995 (PMC7010295; doi:10.1371/journal.pntd.0007995)
Supplement: S5 Table — No trend over time of the male to female ratio was found (z-score of non-parametric test for trend = 0.25). (DOCX) [file pntd.0007995.s006.docx]

**S5 Table:** Absolute numbers of patients diagnosed with visceral leishmaniasis in Kala-Azar Medical Research Center, Muzaffarpur, Bihar, India between 2002-2017. No trend over time of the male to female ratio was found (z-score of non-parametric test for trend = 0.25).

|  |  |  |  |
| --- | --- | --- | --- |
| **Year of reporting** | **Male** | **Female** | **Male to female ratio** |
| 2002 | 162 | 98 | 1.65 |
| 2003 | 62 | 42 | 1.48 |
| 2004 | 358 | 224 | 1.60 |
| 2005 | 406 | 284 | 1.43 |
| 2006 | 287 | 174 | 1.65 |
| 2007 | 268 | 181 | 1.48 |
| 2008 | 416 | 253 | 1.64 |
| 2009 | 283 | 186 | 1.52 |
| 2010 | 368 | 247 | 1.49 |
| 2011 | 72 | 56 | 1.29 |
| 2012 | 14 | 18 | 0.78 |
| 2013 | 110 | 68 | 1.62 |
| 2014 | 208 | 152 | 1.37 |
| 2015 | 245 | 143 | 1.71 |
| 2016 | 183 | 95 | 1.93 |
| 2017 | 85 | 55 | 1.55 |
| **Overall** | **3,527** | **2,276** | **1.55** |
|  |  |  |  |
