# Supplementary material for: Male predominance in reported Visceral Leishmaniasis cases: Nature or nurture? A comparison of population-based with health facility-reported data
Source: PLoS Negl Trop Dis. 2020 Jan 29;14(1):e0007995. doi: 10.1371/journal.pntd.0007995 (PMC7010295; doi:10.1371/journal.pntd.0007995)
Supplement: S6 Table — 30,533 participants–for whom also a second serological result was available—were included to evaluate seroconversion (Kalanet (2006–2009; n = 12,537) and TMRC (2009–2010 (2012–2013 for ‘new area’); n = 17,996). A Direct Agglutination Test cut-off of ≥ 1:1,600 was used to define seropositivity. Direct Agglutination Test cut-off titer of ≥ 1:1,600 was used to define seropositivity. (DOCX) [file pntd.0007995.s007.docx]

**S6 Table:** Risk ratio of males versus females for seroprevalence at baseline and seroconversion as observed through population-based longitudinal studies (Kalanet (2006-2009 ; n = 13,286) and TMRC (2009-2010 (2012-2013 for new area) ; n = 21,050). 30,533 participants – for whom also a second serological result was available - were included to evaluate seroconversion (Kalanet (2006-2009 ; n = 12,537) and TMRC (2009-2010 (2012-2013 for new area) ; n = 17,996). A Direct Agglutination Test cut-off of ≥ 1:1,600 was used to define seropositivity. Direct Agglutination Test cut-off titer of ≥ 1:1,600 was used to define seropositivity.

|  | **Risk ratio male to female**  **Seroprevalence at baseline DAT ≥ 1:1,600**  **(n = 34,336)** | **Risk ratio male to female Seroconversion**  **DAT ≥ 1:1,600**  **(n = 30,533)** |
| --- | --- | --- |
| **Age group (y)** | **RR (95% CI)** | **RR (95% CI)** |
| 0-14 | **1.01** (0.91 - 1.12) | **0.85** (0.71 – 1.02) |
| 15-29 | **1.27** (1.11 - 1.46) | **0.85 (**0.65 – 1.12) |
| 30-44 | **1.18** (1.04 - 1.35) | **1.17** (0.88 - 1.54) |
| 45-59 | **1.22** (1.05 - 1.43) | **1.02** (0.74 - 1.39) |
| 60+ | **1.32** (1.10 - 1.58) | **0.85** (0.58 - 1.23) |
| **Overall** | **1.16** (1.09 - 1.23) | **0.92 (**0.82 – 1.03) |
